# Immunogenicity and Protective Efficacy of Influenza A DNA Vaccines Encoding Artificial Antigens Based on Conservative Hemagglutinin Stem Region and M2 Protein in Mice

**DOI:** 10.3390/vaccines8030448

**Published:** 2020-08-09

**Authors:** Sergei Bazhan, Denis Antonets, Ekaterina Starostina, Tatyana Ilyicheva, Olga Kaplina, Vasiliy Marchenko, Alexander Durymanov, Svetlana Oreshkova, Larisa Karpenko

**Affiliations:** 1 Theoretical Department, State Research Center of Virology and Biotechnology “Vector”, Koltsovo 630559, Novosibirsk Region, Russia; antonec@yandex.ru; 2 Bioengineering Department, State Research Center of Virology and Biotechnology “Vector”, Koltsovo 630559, Novosibirsk Region, Russia; starostina_ev@vector.nsc.ru (E.S.); forelat@ngs.ru (O.K.); sv_oresh@mail.ru (S.O.); lkarpenko@ngs.ru (L.K.); 3 Department of Zoonotic Infections and Influenza, State Research Center of Virology and Biotechnology “Vector”, Koltsovo 630559, Novosibirsk Region, Russia; ilicheva_tn@vector.nsc.ru (T.I.); marchenko_vyu@vector.nsc.ru (V.M.); gavrilych51@mail.ru (A.D.)

**Keywords:** influenza virus, DNA-vaccine constructs, artificial polyepitope T-cell immunogens, hemagglutinin stem region, M2 protein, cross-protective influenza immunity

## Abstract

Background: Development of a universal vaccine capable to induce antibody responses against a broad range of influenza virus strains attracts growing attention. Hemagglutinin stem and the exposed fragment of influenza virus M2 protein are promising targets for induction of cross-protective humoral and cell-mediated response, since they contain conservative epitopes capable to induce antibodies and cytotoxic T lymphocytes (CTLs) to a wide range of influenza virus subtypes. Methods: In this study, we generated DNA vaccine constructs encoding artificial antigens AgH1, AgH3, and AgM2 designed on the basis of conservative hemagglutinin stem fragments of two influenza A virus subtypes, H1N1 and H3N2, and conservative M2 protein, and evaluate their immunogenicity and protective efficacy. To obtain DNA vaccine constructs, genes encoding the designed antigens were cloned into a pcDNA3.1 vector. Expression of the target genes in 293T cells transfected with DNA vaccine constructs has been confirmed by synthesis of specific mRNA. Results: Immunization of BALB/c mice with DNA vaccines encoding these antigens was shown to evoke humoral and T-cell immune responses as well as a moderated statistically significant cross-protective effect against two heterologous viruses A/California/4/2009 (H1N1pdm09) and A/Aichi/2/68 (H3N2). Conclusions: The results demonstrate a potential approach to creating a universal influenza vaccine based on artificial antigens.

## 1. Introduction

Currently, attenuated (live) and inactivated vaccines based on epidemically important influenza strains are administered for prophylactic use. Influenza virus variability is a serious problem that enables it to evade antigen-specific immunity formed due to a previous infection or preceding vaccination. Consequently, it is necessary to change the influenza vaccine composition every 2 or 3 years. Numerous academics as well as industry research teams are trying to develop universal influenza vaccines to solve this problem (see [1,2,3] for review). This task, however, is extremely challenging and requires new approaches.

Studies are being carried out in several main directions including vaccine development based on conservative proteins or conservative sites of variable surface glycoproteins using reverse genetics [4,5] or obtainment of virus-like particles [2,6,7]. Another approach focuses on vaccines based on recombinant plasmid [8] and virus vectors including those based on vaccinia virus [5,9,10], Newcastle disease virus [11], and adenoviruses [12]. Many experimental vaccines demonstrated positive results in animals; some of them are undergoing preclinical or clinical trials. A lot of problems, however, remain to be solved, particularly, moderate immunogenicity of developing vaccines and their inability to provide protection against a broad range of influenza virus subtypes.

Both humoral and cell-mediated responses contribute to organism protection against the influenza virus [13,14], but the proportion of their contribution is not obvious hitherto.

As for the humoral immune response, hemagglutinin (HA) is the main target of antibodies providing protection against the influenza virus [15]. Moreover, virus-neutralizing antibodies primarily target the most variable globular part of HA, making it easier for the virus to evade the preexisting immunity. At the same time, several naturally occurring anti-HA antibodies were discovered, which demonstrated antiviral activity against antigenically different influenza subtypes, including H1N1, H2N2, H3N2, H5N1, and H9N2 [16,17,18]. These broadly neutralizing antibodies recognize conservative epitopes located in the HA stem. Therefore, when constructing universal B-cell immunogens, a promising approach is based on using conservative fragments of the influenza virus HA stem comprising epitopes capable to induce antibodies to a broad range of influenza virus subtypes [19]. Other antigens, candidates for inclusion into the compound of the universal vaccine can include conservative proteins NP, PB1, and M1 [5], as well as the exodomain of the conservative virus protein M2 (M2e) [20], exposed on the surface of infected cells and virion.

T-cell-mediated immune responses also do have a significant impact on influenza virus reproduction reducing illness severity and mortality both in humans [21,22] and in experimental animal models [23,24]. Moreover, T-lymphocytes (both CD4 + and CD8+) predominantly recognize epitopes of the most conservative virus proteins [25]. It was shown that CD8+ T-lymphocytes recognize and lyse infected cells [26], and CD4 + T-lymphocytes enhance antibodies and CTLs responses, as well as contribute to memory cell formation [27,28].

Immunogens developed on the DNA vaccine platform are promising candidates for the creation of the universal vaccine against influenza virus. DNA vaccines can induce both humoral and cellular immune responses [29]. Their ability to induce protective immunity has been demonstrated in animal models [30]. Candidates for universal influenza virus DNA vaccine are most often created using genes encoding conservative proteins NP, M1, M2, and catalytic subunit of RNA-dependent RNA polymerase (PB1). DNA plasmids encoding individual proteins NP [30] and M2 [31] reduce the viral load and increased survival against lethal infection with heterologous viruses in BALB/c mice. DNA immunization with multiple plasmids encoding M1, NP, and PB1 proteins induces cross-protective immunity against heterologous influenza viruses in mice [5], pigs [32], ferrets [33], and non-human primates [34]. DNA vaccines encoding HA consensus sequences and M2e protein also induce broad cross-protective humoral and cellular immunity [35,36].

Thus, when developing a universal vaccine, antigens should be designed in such a way as to induce antibodies and T-cells targeted at conserved epitopes of influenza virus proteins. To deliver the antigens, it is reasonable to use viral or plasmid vectors due to their ability to induce both humoral and cellular immune responses.

The purpose of this study was to evaluate the immunogenicity and protectivity of DNA vaccine constructions encoding artificial antigens designed from conserved hemagglutinin fragments of influenza A virus H1N1 and H3N2 subtypes, and conservative M2 protein.

## 2. Materials and Methods

### 2.1. Design, Synthesis, and Cloning of the Target Genes

Nucleotide sequences of artificial genes encoding the target antigens AgH1, AgH3, and AgM2 were designed using GeneDesigner software [37]. Reverse translation of amino acid sequences of the designed antigens was conducted as a means to keep their nucleotide sequences as close to that of original viral genes as possible; and when codons were changed either to introduce different amino acid residue or to avoid certain restriction sites, human codon usage frequencies were taken into account. The designed genes were synthesized (Evrogen LLC, Moscow, Russia) and cloned in vector plasmid pcDNA3.1. Eventually, three recombinant plasmids were constructed: p-AgH1, p-AgH3, and p-AgM2. Sequences of cloned genes and plasmid fragments containing the promoter region were verified by Sanger sequencing.

### 2.2. RT-PCR Detection of Target Gene Transcription

Verification of target genes transcription was evaluated in HEK293T cells transfected with plasmids p-AgH1, p-AgH3, and p-AgM2 using Lipofectamine 3000 Reagent (Invirtogen, Carlsbad, CA, USA) according to the manufacturer’s protocol. Cells were cultured in DMEM medium with 10% FBS. Following a 48 hr incubation the cells were harvested and total cell RNA was isolated with a kit for RNA isolation (Promega Corporation, Madison, WI, USA) and carried out reverse transcription using RevertAid H Minus First Strand cDNA Synthesis Kit (Thermo Scientific). The obtained cDNA carried out PCR with the use of specific primers listed in Table 1. PCR products were analyzed by electrophoresis in 1% agarose gel.

### 2.3. Ethics Statement

**Figure 1:**
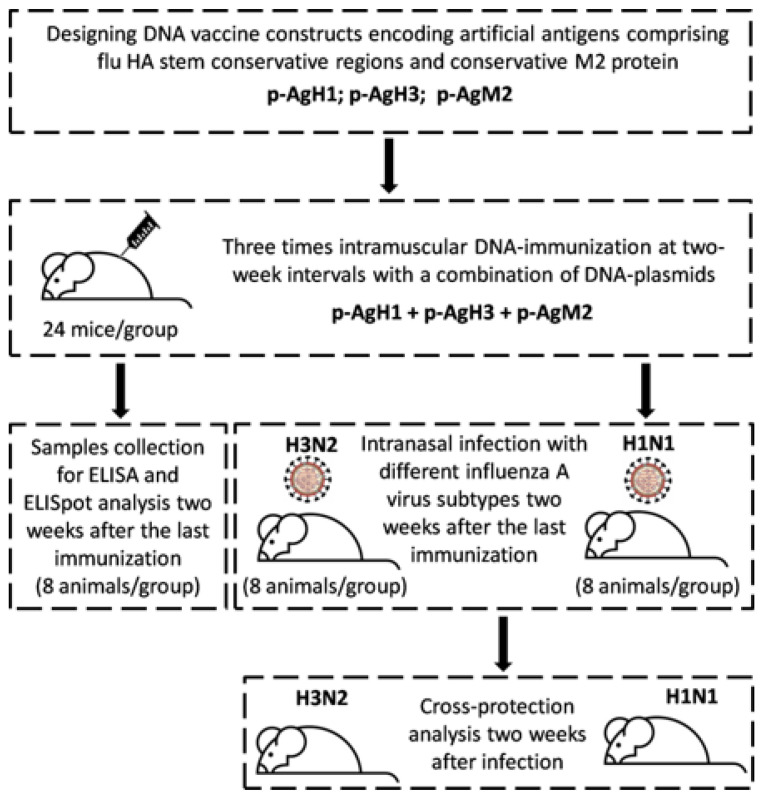
Flow diagram for the conducted animal experiment.

All experimental procedures were conducted in accordance with the recommendations in the “Guide for the Care and Use of Laboratory Animals” (8th edition, 2011). The protocols were approved by the Institutional Animal Care and Use Committee (IACUC) affiliated with “Vector” State Research Center of Virology and Biotechnology (the Permit Number: SRC VB “Vector”/02–05.2016). All efforts were made to minimize animal suffering. Flow diagram for the conducted animal experiment is shown in Figure 1.

### 2.4. Immunization of Experimental Animals and Samples Collection

The immunogenicity of engineered DNA vaccine constructs was evaluated in BALB/c mice. Each experimental and control group of animals consisted of 24 mice. Groups of 5- to 6-week-old female BALB/c mice 16–18 g were immunized three times with intramuscular injections of 100 µg individual plasmid DNA at two-week intervals. In the case of immunization with a mixture of three DNA vaccine constructs, 3 × 75 µg plasmids DNA were injected per mouse. Control mice were immunized with an equivalent dose of empty pcDNA3.1 plasmid DNA. Groups of mice immunized with two strains of influenza virus, either A/California/07/09 (H1N1) or A/Aichi/2/68 (H3N2), were used as positive controls. In this case, the mice were immunized three times with an intramuscular injection of 6 log10 EID50 of each strain in 200 μL of saline. Two weeks after the final immunization, 16 animals from each group were analyzed in challenge studies and 8 animals were used in immunogenic assays. Spleens and blood were harvested from eight animals from each group. Splenocytes were isolated by mechanical disruption and filtration through a 40 μm cell strainer (BD Falcon™). Red blood cells were lysed by treating ACK Lysis Buffer (Sigma), splenocytes were washed in PBS and suspended in RPMI 1640 medium supplemented with 10% FBS plus 100 U/mL penicillin-streptomycin.

### 2.5. ELISpot Assay

Enzyme-Linked ImmunoSpot assay were performed according to the IFN-γ ELISPOT kit protocols (BD Biosciences, San Jose, CA, USA). Briefly, 96-six-well plates were coated with anti-mouse IFN-γ antibody and blocked with RPMI. Splenocytes (2 × 10^5^ cells/well) were plated and stimulated with peptide pool (20 µg/mL of each peptide). The peptides amino acid sequences are listed in Table 2. Plates were incubated for 20 h at 37 °C in 5% CO_2_ before incubation with the detection antibody and development with the AEC substrate solution. The numbers of IFN-γ-producing cells were calculated using an ELISpot-reader (Carl Zeiss, Göttingen, Germany).

### 2.6. Serum ELISA

Antigen-specific serum antibodies were detected by ELISA. Influenza viruses A/Switzerland/9715293/2013 (H3N2), A/Aichi/2/68 (H3N2), and A/California/07/2009 (H1N1pdm09) were used as antigens to assess the specificity and cross-reactivity of an induced humoral response. Ninety-six-well plates (Nunc MaxiSorp, Vienna, Austria) were coated with 50 µL of virus culture medium containing 250 ng of virus proteins, and blocked with 0.5% BSA in phosphate buffered saline (PBS). Sera from individual mice were added at a 1:25 starting dilution, with two-fold serial dilutions in 0.5% BSA. Secondary antibody goat anti-mouse IgG conjugated to horseradish peroxidase (Sigma-Aldrich, Burlington, MA, USA) was added at 1:6000 in 0.5% BSA for one hour. Plates were developed 20 min with TMB (3, 3′, 5, 5′-Tetramethylbenzidine) substrate, and stopped with 2 M sulfuric acid. The OD was measured using a Titertek plate reader at 450 nm.

### 2.7. Challenge Studies

Fourteen days after the last immunization 16 animals from each group were infected intranasally under brief ether anesthesia with 10 LD_50_ of mouse-adapted influenza viruses: 8 mice were challenged with A/California/4/2009 (H1N1pdm09) strain, and the other 8—with A/Aichi/2/68 (H3N2). Following infection, mice were daily monitored over a period of 14 days for their survival.

### 2.8. Statistical Analysis and Software

The differences in quantitative abnormally distributed indicators between independent groups were estimated using a Mann–Whitney test. A comparison between paired groups was carried out with Wilcoxon signed-ranks test. Comparisons between normally distributed values were performed using Welch’s test. FDR-correction was applied for multiple testing. Survival function modeling was conducted using the Kaplan–Meier survival analysis. The differences between survival curves were assessed using the long-rank test (Mantel–Haenszel test).

A sequence analysis was performed using the NIAID Influenza Research Database (IRD) (http://www.fludb.org) [38], and R package Biostrings [39].

Statistical analysis and plotting were executed in R, a programming environment for statistical modeling and analysis (http://r-project.org) [40]. A survival analysis was performed and survival curves were plotted with survival [41] (v. 2.39–5) and survminer [42] (v. 0.3.1) R packages.

## 3. Results

### 3.1. DNA Vaccine Constructions Encoding HA Stem and M2 Protein

Three antigens (Ag) were constructed and used in this study to induce antibodies and T-cell responses. Two of them, AgH1 and AgH3, were designed from conservative fragments of HA stem of H1N1 and H3N2 influenza viruses, respectively, and the third one, AgM2, was a conservative influenza virus M2 protein.

**Figure 2:**
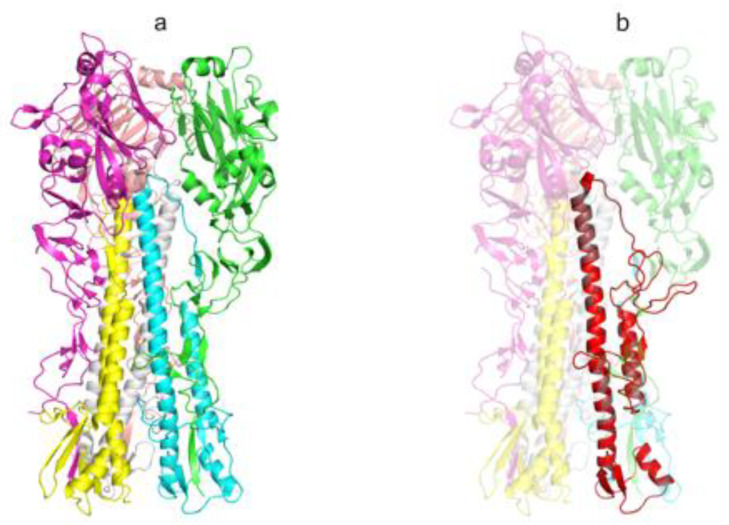
The spatial structure of influenza virus A/Puerto Rico/8/1934(H1N1) HA protein and AgH1 antigen (extracellular portion). (**a**) The structure of the extracellular portion of influenza virus hemagglutinin protein trimer (PDB ID: 1RU7), elements of the secondary structure are shown with the ribbon diagram, individual chains have distinct colors and (**b**) the spatial structure model of artificial AgH1 antigen is shown with red color, the template HA structure is transparent. The picture was produced in PyMOL [45].

Design of AgH1 (H1N1) antigen structure was carried out based on HA of influenza A virus A/Puerto Rico/8/1934(H1N1) as described in [19]. Briefly, the hemagglutinin structure 1RU7 was used as a template (Figure 2a). Antigen structure includes the fragments of HA1 and HA2 subunits forming HA stem and looks as follows: HA1_18-41_–GSA–HA1_290-323_–GSAGSA–HA2_541-613_ (amino acid numbering according to 1RU7 structure).

Amino acid sequence of artificial antigen AgH1 (H1N1) was developed on the basis of influenza virus A/Puerto Rico/8/1934 (H1N1) HA protein according to the algorithm described in [43] with the following exceptions: N-terminal leader peptide was added and a longer HA2 portion was used (separated with a hiphen) with transmembrane and cytosolic fragments (underlined) retained: 

MKANLLVLLCALAAADA-DTVDTVLEKNVTVTHSVNLLEDSHgsaNSSLPYQNTHPTTNGESPKYVRSAKLRMVTGLRNgsagsaTQNAINGITNKVNTVIEKMNIQDTATGKEFNKDEKRMENLNKKVDDGFLDIWTYNAELLVLLENERTLDAHDS-NVKNLYEKVKSQLKNNAKEIGNGCFEFYHKCDNECMESVRNGTYDYPKYSEESKLNREKVDGVKLESMGIYQILAIYSTVASSLVLLVSLGAISFWMCSNGSLQCRICI

Herein MKANLLVLLCALAAADA is the leader peptide; lower-case letters denote amino acid residues corresponding to linker peptides. We also included the following amino acid substitutions into the antigen: I298T, V301T, I303N, V566T, and F610A to reduce hydrophobic nature on the exposed surface; F563D and L573D—to destabilize the structure formed in acid medium; C306S—to evade formation of adverse disulfide bonds as it was done in [19]; and mutations S554T and N582K were added to make an antigen sequence closer to the most common sequence variant. Amino acids are numbered according to 1RU7 structure. Using Modeller software [44] we built a model of the AgH1 spatial structure (Figure 2b).

Transmembrane and cytosolic fragments were retained in the final antigenic construction to allow surface exposure of the target antigen on virus-like particles, or on the surface of cells transfected with DNA vaccine plasmid encoding this antigen.

An artificial antigen AgH3 (H3N2) was developed in a way similar to the AgH1 (H1N1) design. Its primary structure, however, was designed as a consensus sequence obtained from alignment of HA proteins of several influenza vaccine strains of H3N2 subtype: A/Switzerland/9715293/2013, A/HongKong/4801/2014, A/Wisconsin/67/2005, A/Brisbane/10/2007, A/Perth/16/2009, A/Victoria/361/2011, A/Texas/50/2012, and two additional H1N1 strains: A/Puerto Rico/8/1934 and A/California/7/2009. The alignment was built using NIAID Influenza Research Database (IRD) web-portal analytical tools (http://www.fludb.org) [38]. The AgH3 antigen amino acid sequence looks as follows: 

MKTIIALSYILCLVFAQ-TIVKTITNDQIEVTNATELVQSSSgsaPNDKPFQNVNRITYGASPRYVKQNTLKLATGMRNgsagsaTQAAINQINGKLNRLIGKTNEKDHQIEKEFSEDEGRIQDLEKYVEDTKIDLWSYNAELLVALENQHTIDLTDS-EMNKLFERTKKQLRENAEDMGNGCFKIYHKCDNACIGSIRNGTYDHDVYRDEALNNRFQIKGVELKSGYKDWILWISFAISCFLLCVALLGFIMWACQKGNIRCNICI.

MKTIIALSYILCLVFAQ is a consensus sequence of the leader peptide; lower case letters denote amino acid residues corresponding to linker peptides. Sequences of artificial genes encoding antigens AgH3 and AgH3 were obtained using reverse translation in GeneDesigner (DNA2.0 Inc., Menlo Park, CA, USA) [37] based on natural nucleotide sequences (NC_002017.1, EF473465.1, and KM821341.1).

**Figure 3:**
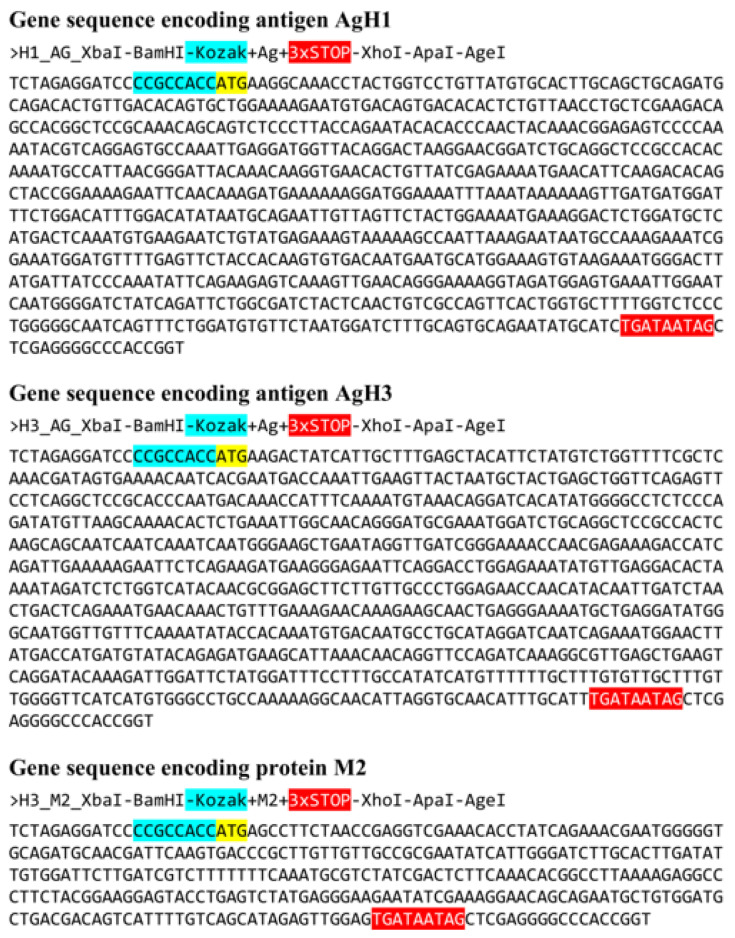
Nucleotide sequences of developed antigens. Kozak sequences are marked with cyan color. Start codons are marked with yellow. Stop codons highlighted with red. Each gene is flanked with restriction sites: 5′-end: XbaI and BamHI sites; 3′-end: XhoI, ApaI, and AgeI sites.

An analysis of amino acid sequences of M2 proteins demonstrates that its exposed fragment is highly conservative and remains unchanged in all influenza virus strains listed above. The nucleotide sequence of artificial gene encoding M2 protein (AgM2) was based on the 7th segment of virus A/Wisconsin/67/2005(H3N2) (EU100611.1). The final sequences of all artificial genes encoding target antigens AgH1, AgH3, and 2 are shown in Figure 3.

Designed genes were synthesized (Evrogen LLC, Russia) and cloned into pcDNA3.1. Three recombinant DNA vaccine plasmids were produced: p-AgH1, p-AgH3, and p-AgM2.

### 3.2. Verification of Target Genes Transcription in Transfected Cells

Eukaryotic 293T cells were transfected with DNA vaccine constructs p-AgH1, p-AgH3, and p-AgM2 comprising sequences of the target genes and target genes expression were confirmed with corresponding mRNA detection using RT-PCR as described in the Materials and Methods section.

**Figure 4:**
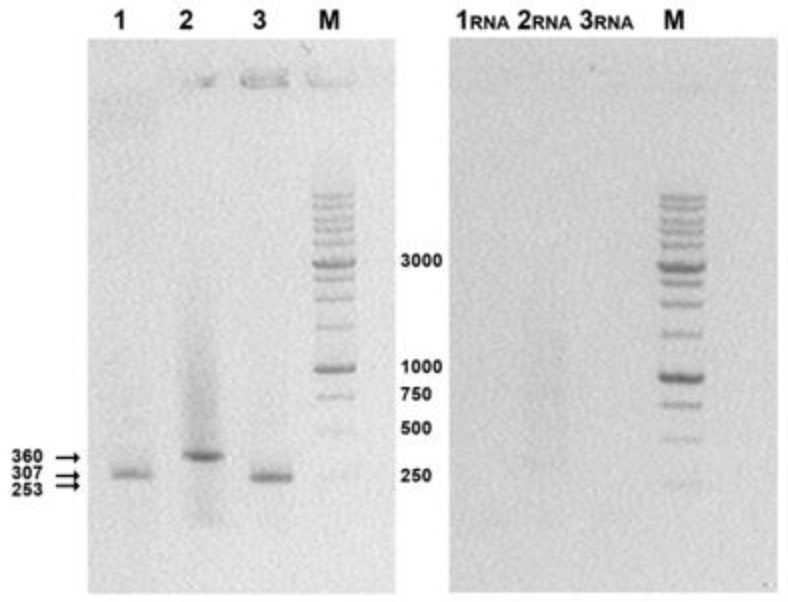
Electrophoretic analysis of RT-PCR products in 1% agarose gel. 1, 2, 3, RT-PCR products were obtained using cDNAs from 293T cells transfected with the target plasmids: 1, p-AgH1; 2, p-AgH3; 3, p-AgM2; 1_RNA_, 2_RNA_, and 3_RNA_, PCR reaction in these probes was performed without reverse transcription of RNAs isolated from transfected 293T cells (this was done to check both primers specificity and the lack of plasmids admixtures); M, M12 oligonucleotide size markers produced by SibEnzyme (DNA fragments 10000, 8000, 6000, 5000, 4000, 3000, 2500, 2000, 1500, 1000, 750, 500, and 250 bp).

Electropherograms (Figure 4) show that the sizes of the amplified fragments correspond to the theoretically calculated product lengths of the target genes: 307 bps for AgH1, 360 bps for AgH3, and 253 bps for AgM2. Thus, the obtained data demonstrate the presence of specific mRNA in total RNA samples extracted from transfected cells and thus confirm the expression of the target genes.

### 3.3. Immunogenicity of DNA Vaccine Constructs Encoding the Target Antigens

Female BALB/c mice weighting 16–18 g were used for immunization. All animals were divided into seven groups with eight mice in each group:

- AgH1—mice were immunized with DNA-plasmid p-AgH1;
- AgH3—immunized with DNA-plasmid p-AgH3;
- AgM2—immunized with DNA-plasmid p-AgM2;
- Ag(H1 + H3 + M2)—immunized with a mixture of p-AgN1, p-AgN3, and p-AgM2 DNA-plasmids;
- Ag0—immunized with empty vector plasmid pcDNA3.1 (negative control);
- Intact—nonimmunized mice (negative control);
- H1N1pdm09—mice immunized with influenza A virus strain A/*California*/*07/2009* (H1N1pdm09; positive control).

Mice were immunized three times at two-week intervals by intramuscular injections of 100 µg DNA. Blood samples and splenocytes were collected two weeks after the 3rd immunization. Blood samples were used to estimate B-cell responses in ELISA assays and splenocytes to assess T-cell responses using the IFN-γ-ELISpot assay. The A/California/07/09 (H1N1pdm09) influenza virus strain was used as a target antigen in ELISA.

**Figure 5:**
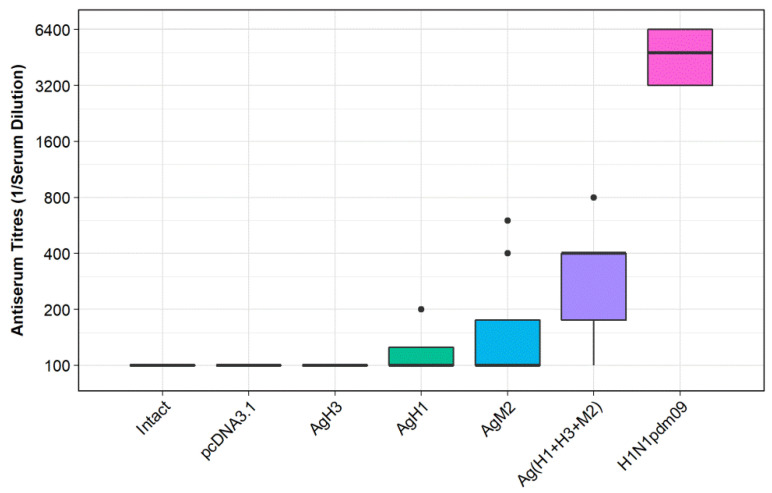
Serum antibody titers observed in BALB/c mice immunized with DNA vaccine constructs encoding the antigens. AgH1, mice immunized with DNA-plasmid p-AgH1; AgH3, mice immunized with DNA-plasmid p-AgH3; AgM2, mice immunized with DNA-plasmid p-AgM2; Ag(H1+H3+M2), mice immunized with a mix of DNA-plasmids p-AgH1+p-AgH3+p-AgM2; pcDNA3.1, mice immunized with vector plasmid (negative control); intact, nonimmunized mice (negative control); and H1N1pdm09, mice immunized with A/California/07/2009 (H1N1pdm09) virus strain (positive control).

Figure 5 represents ELISA results. The analysis of obtained results demonstrated that the titers of antibodies (induced by each target construct individually) did not differ from those obtained from the negative controls: groups Ag0 and Intact. Immunization with combination of DNA vaccines, group Ag(H1 + H3 + M2), was found to be more efficient. In this case the antibody titers significantly exceeded the values observed both in the negative controls: Ag0 (*p* < 0.035) and Intact (*p* < 0.007), and in two experimental groups: AgH1 (H1N1; *p* < 0.0232) and AgH3 (H3N2; *p* < 0.009). Immunization with virus H1N1pdm09 (positive control groups), however, was far more effective. In this case the antibody titers significantly exceeded the values observed in all experimental and negative control groups (*p* < 0.0081).

Thus, the obtained results revealed that a combination of DNA vaccine constructs encoding artificial antigens designed from conservative fragments of HA stem of influenza viruses H1N1 and H3N2, and M2 protein can induce a statistically significant antibody response towards the influenza virus in immunized mice.

To evaluate T-cell responses in the IFN-γ-ELISpot assay, splenocytes isolated from immunized mice were stimulated with a mixture of peptides representing potential CB8+ T-cell epitopes restricted by MHC class I (H2-D^d^, H2-L^d^, and H2-K^d^) alleles of BALB/c mice. The peptides are listed in Table 2. MHC binding predictions were conducted using NetMHC [46] and NetMHCIIpan web-servers [47].

A comparison of T-cell responses in different groups of immunized BALB/c mice showed that immunization with a combination of DNA vaccines Ag(H1 + H3 + M2) induced the highest specific T-cell response comparable to that in the positive controls. In other experimental groups, the level of T-cell immune responses was low and did not exceed that of the negative controls—Intact and Ag0 (pcDNA3.1). A statistical analysis of the findings performed using one-way Welch’s test with FDR-correction revealed that significant results were observed only in two groups: in the animals immunized with a combination of DNA vaccines (*p* < 0.05) and the animal group immunized with vaccine strain A/California/07/2009 (H1N1pdm09; *p* < 0.0038).

Consequently, immunogenicity studies of DNA vaccine constructs demonstrated that our designed antigens can induce both humoral and cellular immune responses in immunized mice, if used in combination.

### 3.4. Protective Efficacy of the Developed DNA Vaccine Constructs

The protective effect of the developed DNA vaccine constructs was studied in experimental intranasal infection with lethal virus dose (10 LD_50_). In challenged experiments we used only a group of mice immunized with a mixture of three DNA vaccine constructs (p-AgH1, p-AgH3, and p-AgM2). Mice immunized with individual plasmids were not analyzed since only a combination of developed DNA vaccines was found to induce significant immune responses. Mice were challenged 14 days after the 3rd immunization with one of two mouse-adapted influenza viruses: Influenza viruses A/California/4/2009 (H1N1pdm09) and A/Aichi/2/68 (H3N2) strains.

The protectivity of the target DNA vaccine constructs was studied in four animal groups including:

- Ag(H1 + H3 + M2)—mice immunized with a combination of DNA-plasmids p-AgH1+p-AgH3+p-AgM2 (75 µg/mice for each plasmid);
- H1N1—mice immunized with A/*California*/*07/2009* (H1N1pdm09) virus (positive control);
- H3N2—mice immunized with A/Aichi/2/68 (H3N2) virus strain (positive control);
- pDNA3.1—mice immunized with empty vector plasmid pDNA3.1 (negative control; 200 µg/mice).

Intact nonimmune mice were used to control the infective doses of viruses in the study.

A survival analysis was carried out with Kaplan–Meier analysis and survival curves were compared using the long-rank test (Mantel–Haenszel test).

**Figure 6:**
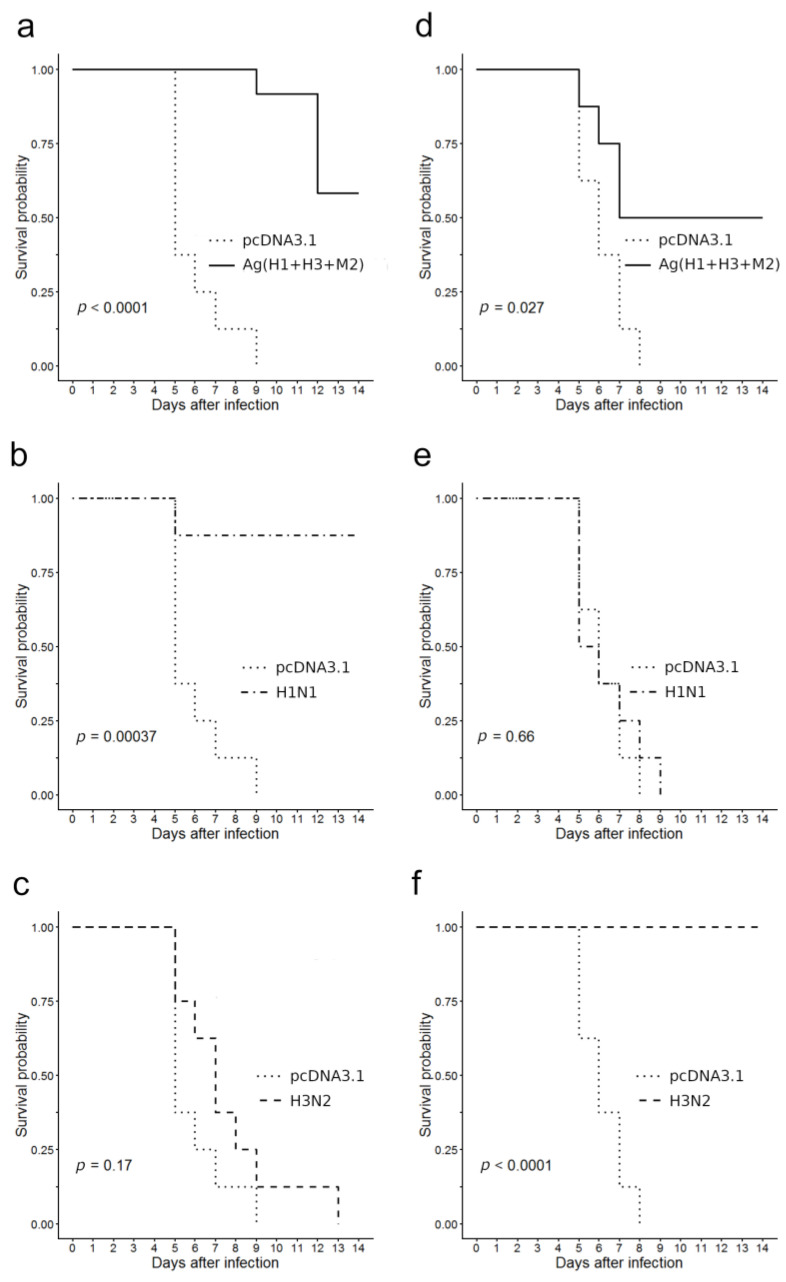
Immunized mice survival after a lethal challenge with pathogenic influenza A viruses in comparison to that of the negative control (pcDNA3.1). (**a**–**c**) Infection with virus A/California/4/09 (H1N1pdm); (**d**–**f**) infection with virus A/Aichi/2/68 (H3N2); (**a**,**d**) DNA vaccinated, (**b**) and (**e**) H1N1 vaccinated, and (**c**,**f**) H3N2 vaccinated.

Figure 6 summarizes the survival data. The findings revealed that a statistically significant protective effect against lethal challenge with the A/California/4/2009 virus was registered in two groups of immunized mice, namely in the group of mice H1N1 immunized with A/California/07/09 (H1N1pdm09; *p* = 0.00037, Figure 6b) and in the group of mice Ag(H1 + H3 + M2) immunized with a combination of DNA-plasmids (*p* = 0.0001, Figure 6a). The survival rate in group H1N1 was 87.5% and in group Ag(H1+H3+M2)—58.3%. In group immunized with A/Aichi/2/68 (H3N2) virus all the animals died; there was no statistically significant differences in their survival curves from that of negative control immunized with empty vector pcDNA3.1 (*p* = 0.17, Figure 6c).

In the case of lethal infection with the A/Aichi/2/68 (H3N2) virus, the protective effects were registered in animals, immunized with the A/Aichi/2/68 (H3N2) virus (all eight animals survived, *p* < 0.0001, Figure 6f) and in animals, immunized with a mixture of DNA vaccines (four out of eight mice survived, *p* = 0.027, Figure 6d). In groups immunized with either an empty vector plasmid or with an influenza virus of the H1N1 subtype all animals died 8–9 days after infection (*p* = 0.66, Figure 6e). The difference between the survival curve of the negative controls and that of the Ag(H1 + H3 + M2) group was found to be statistically significant (*p* = 0.027, Figure 6d).

Thus, our findings demonstrate that mice immunization with a combination of DNA vaccine constructs encoding artificial antigens designed from two variants of influenza HA proteins stem regions and conservative M2 protein stimulated cross-protective immunity both against the A/Aichi/2/68 (H3N2) strain and the A/*California*/*07/2009* (H1N1pdm09) strain (Figure 6a,d).

We also studied humoral and T-cell responses in immunized animals to understand the reasons behind the obtained results.

The results of B-cell response studies shown in Table 3 illustrated that immunization with a mixture of DNA vaccine constructs encoding target antigens induced antibodies towards both the A/Aichi/2/68 (H3N2) and A/California/07/2009 (H1N1pdm09) influenza virus strains. Antibodies recognizing A/Aichi/2/68 and A/California/07/09 were detected in all eight mice immunized with p-AgH1+p-AgH3+p-AgM2 DNA-plasmids combination.

**Figure 7:**
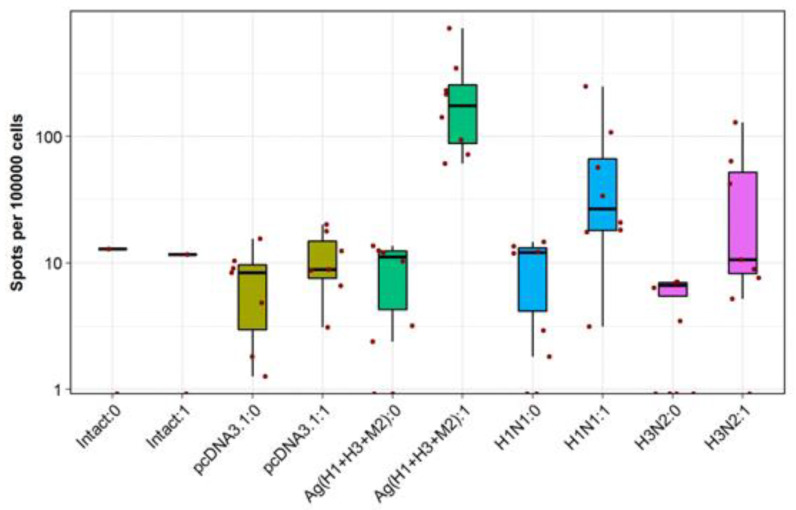
Evaluation of cellular responses in immunized mice using the IFN-γ-ELISpot assay. Distinct colors designate the results observed in different experimental and control groups. “0” in the name of a group corresponds to a spontaneous IFN-γ secretion, and “1” corresponds to IFN-γ secretion stimulated with antigenic peptides. Cell counts are presented on a log scale (10 base).

These DNA vaccines used together were also found to induce high rates of IFN-γ-producing T-cells in response to stimulation with specific peptides in IFN-γ-ELISpot assays (Figure 7). Only splenocytes of Ag(H1 + H3 + M2) group mice demonstrated a statistically significant induction of antigen-specific IFN-γ secretion, exceeding that of splenocytes from both the negative controls and the animals immunized with live viruses. A higher IFN-γ secretion observed in the last case can be explained by incomplete correspondence between the peptides, selected from artificial antigens AgH1, AgH3, and AgM2 to stimulate splenocytes, and the amino acid sequences of viral antigens.

Thus, the obtained results suggest that both humoral and cellular immune responses contributed to cross-protection against lethal infection with A/California/4/09 (H1N1pdm) and A/Aichi/2/68 (H3N2) strains in mice vaccinated with a mixture of pAgH1, pAgH3, and pAgM2 plasmids.

## 4. Discussion

Influenza is a highly contagious acute respiratory disease caused by influenza viruses. Yearly seasonal influenza outbreaks all around the world affect 5–15% of the human population and cause significant mortality among the risk groups as well as have a serious economic impact. To undercut influenza impacts, there are effective prophylactic influenza vaccines made of epidemically significant attenuated live or inactivated virus strains. The high variability of influenza viruses, however, makes the viruses evading the pre-existing immunity, and consequently, the influenza vaccine composition should be changed every 2 or 3 years to match the actual circulating strains. Thus, developing a universal influenza vaccine capable to induce protective immunity against the widest possible range of influenza virus strains has attracted increasing attention. Over the last decade, remarkable progress has been made in this area through studies of broadly neutralizing antibodies targeted at the conservative stem region of HA proteins; experimental vaccines, based on the influenza virus conservative M2 protein surface exposed fragment (M2e); and with numerous experimental vaccines constructions based on conservative viral proteins and their fragments [16,19,20,48,49].

The main aim of the current study was to develop artificial vaccine constructs based on conservative fragments of the HA stem and conservative M2 protein of influenza A virus, and to evaluate their immunogenicity and protectivity against influenza viruses of different subtypes in a mouse model.

As is known, vaccine efficacy depends not only on its composition, but also on the method of antigen delivery and presentation to T- and B-lymphocytes. In our study, the antigens designed were used in the form of DNA vaccine plasmids, injected intramuscularly, since DNA vaccines were shown to induce both humoral and cellular immune responses towards the products encoded. Moreover, DNA vaccination is one of the most natural ways to stimulate a cytotoxic CD8+ T-cell response.

Evaluation of the immunogenic properties of the designed DNA vaccine constructs was carried out in BALB/c mice. Stimulation of T-cell responses was assessed with the IFN-γ-ELISpot assay, and serum titers of specific antibodies were studied with ELISA using two influenza A virus strains belonging to two different subtypes (H3N2 and H1N1) as the targets. The protectivity of the designed vaccine constructs was also tested in immunized animals against lethal infection with viruses A/Aichi/2/68 (H3N2) and A/California/4/2009 (H1N1pdm09). These studies demonstrated that only immunization with a combination of three vaccine plasmids resulted in significant stimulation of both humoral and T-cell antigen-specific responses in BALB/c mice. Inability of designed vaccine plasmids to stimulate immune responses when used separately can suggest that their combined administration have a synergistic effect.

Since statistically significant immune responses were detected only in animals immunized with a combination of DNA vaccine plasmids, only the protectivity of combined immunization was studied and the protectivity of individual vaccine constructions was not tested. Immunization with a combination of DNA vaccines provided moderated statistically significant cross-protection in immunized mice against lethal infection with 10 LD_50_ both A/California/4/2009 (H1N1pdm09 and A/Aichi/2/68 (H3N2) viruses—58.3% and 50%, respectively (Figure 6a,d).

To understand the reasons behind these results, we studied specific humoral and cellular responses in immunized animals (Table 3, Figure 7). The findings suggest that both antibody and T-cell responses can contribute to cross-protection of immunized mice against lethal infection with both A/California/4/2009 (H1N1pdm09 and A/Aichi/2/68 (H3N2) viruses.

In general, the obtained results reveal that the designed DNA vaccine constructs provide target gene expression (Figure 4); and administered in combination they induce significant levels of specific antibodies (Table 3) and T-cell response (Figure 7), and provide cross-protection against the two analyzed virus strains (Figure 6a,d). The antibodies were shown to recognize both H1N1 and H3N2 viruses (Table 3). Besides, the level of T-cell responses in mice immunized with a mixture of the target DNA vaccine plasmids significantly exceeded those of animal immunized with live viruses (Figure 7). This result, however, can be also explained by incomplete correspondence between these peptides and amino acid sequences of viral antigens, since the peptides used in ELISpot for antigenic stimulation were selected from artificial antigens AgH1, AgH3, and AgM2.

Along with that, our findings demonstrated a relatively weak protective effect of the obtained vaccine constructions that was likely associated with insufficient levels of specific antibodies and CTLs induced by vaccination. This was not unexpected since immunization with naked DNA is known to induce poor or modest immunogenicity, therefore, we plan to use in vivo electroporation in upcoming experiments, as it was shown to achieve almost a 1000-fold increase in DNA vaccines immunogenicity [50,51], and we also work to produce virus-like particles based on the developed target antigens. Currently, virus-like particles are actively used in vaccine development including the universal influenza vaccine [2,6,7].

When designing vaccine constructs, we used conservative fragments of HA and protein M2. It was shown that many antibodies to conservative sites of internal proteins lack neutralizing activity in vitro [52]. In our case antibodies from sera of mice immunized with the obtained DNA vaccine constructs failed to show neutralizing activity in vitro, too (data not presented), although they interacted with H1N1 and H3N2 virus antigens in ELISA (Table 3). Nevertheless, it was demonstrated that non-neutralizing antibodies to the influenza virus can result in inhibition of infectious process in vivo blocking fusion of viral and endosomal membranes [53], or induce complement-mediated lysis of the infected cells and antibody-dependent cell cytotoxicity (ADCC) [54]. A potential role of non-neutralizing antibodies has also been shown against HIV-1 in RV144 clinical trial; their protective effect may be mediated by ADCC [55].

## 5. Conclusions

This study implements one of the possible approaches to creating a universal influenza vaccine based on engineering artificial antigens made of conservative hemagglutinin stem regions and conservative M2 protein. The obtained results show that the designed artificial antigens have been expressed from corresponding artificial genes cloned into DNA plasmids. It is also demonstrated that DNA vaccination with plasmids encoding target antigens evokes both specific antibodies and T-cell responses against two analyzed virus strains A/California/4/09 (H1N1pdm09) and A/Aichi/2/68 (H3N2) in immunized mice and provides cross-protection animals against the lethal challenge with these strains. In future we plan to use in vivo electroporation as well as virus-like particles to increase the immunogenicity and protectivity of developed antigens.

## Figures and Tables

**Table 1:** Primers used to detect synthesis of the target mRNAs in RT-PCR.

Primers	Annotation
F: 5′-ACTGTTGACACAGTGCTGGAAAAGAAT-3′ R: 5′-TTTTCATCTTTGTTGAATTCTTTTCC-3′	Primers pair to detect mRNA encoding antigen AgH1
F: 5′-GCTTTGAGCTACATTCTATGTCTGG-3′ R: 5′-GGTCCTGAATTCTCCCTTCATCTTC-3′	Primers for detection of AgH3 mRNA
F: 5′-GAATGGGGGTGCAGATGCAACGATTC-3′ R: 5′-CAACTCTATGCTGACAAAATGACTGTC-3′	Primers for AgM2 mRNA detection

**Table 2:** Peptides selected for splenocytes stimulation in IFN-γ-ELISpot assays.

Peptides in Composition of the Designed Antigens		
AgH1	AgH3	AgM2
KYVRSAKLR	LFERTKKQL	RGPSTEGVP
LYEKVKSQL	HDVYRDEAL	ETPIRNEWG
FYHKADNEA	RYVKQNTLK	LLTEVETPI
AKLRMVTGL	KPFQNVNRI	
SHGSANSSL	LENQHTIDL	
	KSGYKDWIL	
	IEVTNATEL	

**Table 3:** Titers of anti-influenza virus antibodies assessed with ELISA in immunized mice sera.

**Antigen/Number of Mice/Reverse Titer in ELISA against A/California/07/09 (H1N1)**												
Ag(H1 + H3 + M2)								H1N1	H3N2	pcDNA		
1	2	3	4	5	6	7	8	1	1	1	2	3
1600	1600	1600	1600	3200	3200	3200	3200	12,800	3200	200	200	200
**Antigen/Number of mice/Reverse Titer in ELISA against A/Aichi/02/68 (H3N2)**												
Ag(H1 + H3 + M2)								H1N1	H3N2	pcDNA		
1	2	3	4	5	6	7	8	1	1	1	2	3
1600	3200	1600	3200	6400	6400	3200	6400	6400	12800	200	400	400
